# Incorporation of Silica Particles Attached to Nylon 66 Electrospun Nanofibers with Cement

**DOI:** 10.3390/ma15197011

**Published:** 2022-10-10

**Authors:** Tri N. M. Nguyen, Do Hyung Lee, Jung J. Kim

**Affiliations:** 1 Campus in Ho Chi Minh City, University of Transport and Communications, No. 450-451 Le Van Viet Street, Tang Nhon Phu A Ward, Thu Duc City, Ho Chi Minh City 700000, Vietnam; 2 Department of Civil, Railroad and Unmanned Systems Engineering, PaiChai University, 155-40 Baejaero, Seo-gu, Daejeon 35345, Korea; 3 Department of Civil Engineering, Kyungnam University, 7 Kyungnamdaehakro, Changwon-si 51767, Korea

**Keywords:** nylon 66, silica, electrospun nanofibers, cement, mechanical strength, microstructure

## Abstract

In this study, a modified version of electrospun nylon 66 nanofibers by silica particles were blended into ordinary Portland cement to investigate the microstructure and some mechanical properties of cementitious material. The addition of silica into the nanofibers improved the tensile and compressive properties of the hardened cement pastes. The observations from the mechanical strength tests showed an increase of 41%, 33% and 65% in tensile strength, compressive strength, and toughness, respectively, when modifying the cement pastes with the proposed nanofibers. The observations from scanning electron microscopy and transmission electron microscopy showed the morphology and microstructure of the fibers as well as their behaviors inside the cement matrix. Additionally, X-ray diffraction and thermal gravimetric analysis clarified the occurrence of the extra pozzolanic reaction, as well as the calcium hydroxide consumption by the attached silica inside the cement matrix. Finally, the observations from this study showed the successful fabrication of the modified nanofibers and the feasibility of improving the tensile and compressive behaviors of cement pastes using the proposed electrospun nanofibers.

## 1. Introduction

Today, cement is a basic material that plays an important role in the construction sector. However, there is an increased demand for higher cement strength, due to the feasibility of reducing structural size and cement consumption, which can lead to significant ecological improvement and financial efficiency. Therefore, it has posed a considerable challenge for researchers and manufacturers. Cement matrix strength can be affected by different factors, such as the component ratio, the cement hydration process, the change in the cement matrix microstructure, the curing condition, the addition of additives, and so on [1,2]. Traditionally, silica has been used as a supplement to increase the strength of cementitious materials. The small and fine silica particles provide the advantage of filling up the porous structure of the matrix, leading to the formation of a compact structure. In addition, from a chemical perspective, silica particles are a highly reactive mineral with the ability to participate in the pozzolanic reaction with calcium hydroxide (CH) generated from the cement hydration process [3,4,5,6,7]. Consequently, calcium silicate hydrate (CSH) is formed, which, as demonstrated, comprises approximately 60% of the solid volume of the hydration product, and is crucial in improving the long-term strength and durability of the cement matrix [1]. A study on the effect of nanosilica (NS) on the compressive strength of cement paste reported a maximum increase of 43.8% at 0.6% NS by weight of cement [8]. In addition, Flores et al. [9] reported an increase of 25% in the compressive strength of pastes containing NS or silica fume compared to that of plain paste when replacing cement with an additive proportion of 2.5% by mass. They also concluded that these supplementary factors influence the acceleration of the hydration reactions in the early stages. Mohammed et al. [10] reported an increase from 15% to 50% in the compressive strength of cement pastes containing 1% NS depending on the curing time and water-to-binder ratio.

Furthermore, recent findings showed significant performance of different nanosized fibers in improving the cement matrix. The findings by Liu et al. [11] showed the effectiveness of nanocarbon black and nickel nanofiber in improving the electrical and piezoresistive behaviors of cement. Therefore, the result shows the feasibility of utilizing that kind of cement composite in the sensor industry. The study by Haider et al. [12] reported the influence of chitin nanofiber on delaying setting time and increasing viscosity. Based on those findings, chitin nanofiber can become a promising candidate for tailoring cement for specific applications. From a mechanical strength perspective, the study [12] reported that after 28 days, the compressive strength and the flexural strength of the cement paste containing chitin nanofiber increased by 12% and 41%, respectively, compared to that of the plain paste. In addition, the role of carbon nanofiber in improving Young’s modulus of cement paste by 15.5% compared to that of plain paste was reported [13]. The study [14] also reported an improvement of 18% in terms of compressive strength and 21% in terms of flexural strength when introducing cellulose nanofibers into the cement paste. Alternatively, a new approach to strengthen ordinary Portland cement (OPC) using electrospun nanofibers has been suggested in previous works [15,16,17,18]. The work [16] presented an approach to reinforce cement materials using electrospun nylon 66 nanofibers, followed by blending the as-spun nanofibers into cement. The results showed the bridging effect of the nylon 66 nanofibers inside the matrix. Furthermore, the increase in tensile strength of up to 30% confirmed the feasibility of this approach for enhancing cement characteristics. Continuing this strategy, the present study focuses on investigating the influence of electrospun nylon 66 nanofibers modified with silica particles (nanosilica) (NS-N66 NFs) on the mechanical characteristics of cement and its microstructural properties. The as-spun nanofibers were blended into the cement powder using an improved collector kit. Tensile strength and compressive strength tests were conducted to examine the changes in the mechanical characteristics of OPC. In addition, microstructural analysis methods, such as field emission scanning electron microscopy (FE–SEM), field emission transmission electron microscopy (FE–TEM), X-ray diffraction (XRD), and thermal gravimetric analysis (TGA), were employed to analyze the microstructural characteristics of the modified nanofibers and the nanofiber-blended cement pastes.

## 2. Materials and Methods

### 2.1. Materials

**Figure 1:**
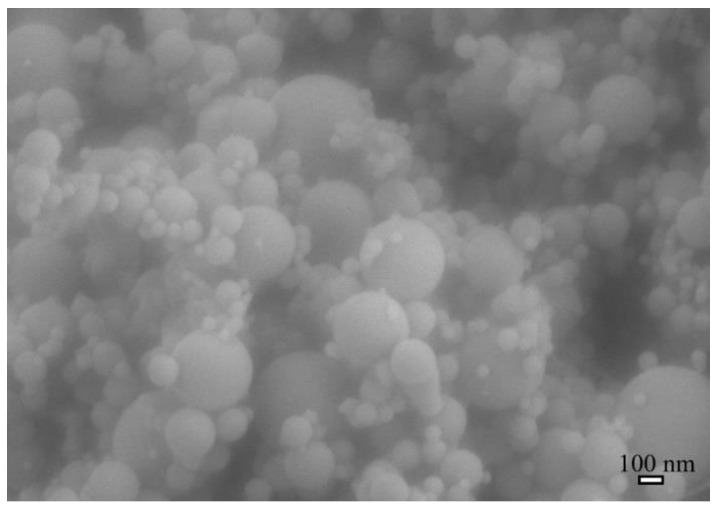
Morphology of silica particles.

Type I ordinary Portland cement was purchased from Ssangyong Co., Korea [19]. The properties of the cement are listed in Table 1. Nanosilica grade 940-U (CAS No. 69012-64-2) was purchased from Elkem Microsilica (Norway). Figure 1 presents the morphology of the silica particles and their diameter distribution, which ranges from 30 nm to 510 nm, with a mean diameter of approximately 120 nm. Nylon 66 pellets and other chemicals for its dissolution (formic acid and chloroform) were prepared as previously described [15,16]. All materials were used as received.

### 2.2. Polymer Solution

The polymer solutions were prepared in two steps: (1) The solvent was prepared by stirring formic acid and chloroform in a volume ratio of 4:1. (2) Precursors, silica particles, and nylon 66 were added to the prepared solvent to obtain the polymer solutions at a precursor-to-solvent ratio of 1:4 by weight of the polymer solution. This proportion was determined after conducting a trial-and-error process to determine the optimal and spinnable viscosity of the polymer solution using a size 20 Taylor cone. To be more specific, 13 wt% of silica was added to two bottles containing 80 wt% of solvent. Then, the solutions were ultrasonicated for 2 h under laboratory conditions to break the van der Waals forces between the nanomaterial molecules and obtain homogeneous dissolution in the final solutions [20,21,22]. Finally, 7 wt% of nylon 66 was added to the solution and stirred for 3 h to obtain homogeneous polymer solutions. Due to the relaxation of the polymer chain, the polymer solutions were kept for 24 h under laboratory conditions before conducting the electrospinning process.

### 2.3. Electrospinning Process, Hardened Cement Paste Preparation, and Testing Methods

Electrospun nanofiber fabrication and the blending process to obtain the composite cementitious materials containing electrospun nanofibers have been reported in previous works [15,16]. After jetting out from the needle, the nanofibers are blended into cement by an improved collector as a single fiber, and the nanofiber length is broken during the blending process. As in those studies, we retained all of the input parameters of the electrospinning process and the electrospun nanofiber proportion of 5% of the cement mass here as well.

Owing to the changes in the microstructure of the matrix and the difficulty in controlling the spontaneous cracking that occurs during the curing process, it is difficult to conduct a study on the mechanical properties of hardened cement pastes. Therefore, the cement paste samples were designed with small dimensions according to the specifications in the ASTM C307 [23] and ASTM C 109/109M [24] methods for conducting tensile strength and compressive strength tests, respectively. Five briquette samples and five cubic samples for each cement composite were prepared with a constant water-to-binder proportion of 0.5 [25]. Then, the samples were cured in water at an ambient temperature of 23 ± 2 °C and a relative humidity of 50% for 28 days prior to testing.

In this study, the tensile strength and compressive strength tests were evaluated using a 5 kN mortar tensile strength test device in compliance with ASTM C307 and a 1000 kN hydraulic universal testing machine in compliance with ASTM C109/109M, respectively.

The FE–SEM analysis was conducted using a Zeiss Merlin Compact system. Input parameters, a working distance of 9 mm, and an acceleration voltage of 5 kV were used. In addition, to increase the observation quality, a 5 Å platinum cover was coated on the sample surfaces.

The FE–TEM analysis was conducted using an FEI Tecnai F30 Twin system under an acceleration voltage of 300 kV.

XRD analysis was conducted using the D8 Advance system with a scanning speed of 0.4 s/step, a step size of 0.02° (2θ)/step from 5° to 70°, and CuKα radiation (40 kV, 40 mA).

TGA was conducted using a TA instrument SDT-Q600 under a nitrogen atmosphere with a flow rate of 100 mL/min, a heating velocity of 10 °C/min, and a heating range from 25 °C to 1000 °C.

## 3. Results and Discussion

### 3.1. Mechanical Strength

**Figure 2:**
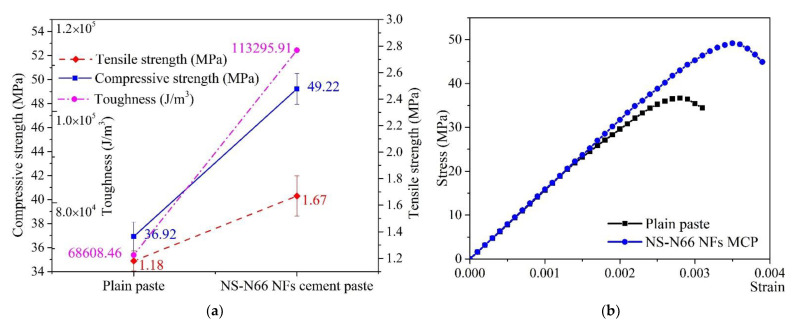
(**a**) The mechanical behaviors of the cement pastes at 28 days; (**b**) The constitutive curves, where “MCP” implies “modified cement paste”.

The results of the tensile and compressive strength tests indicate that the electrospun nylon 66 nanofibers modified with silica particles are effective in improving some select mechanical properties of the hardened cement paste (see Figure 2). Overall, the results showed an upward trend in the mechanical strength of the cement blended with NS-N66 NFs. Compared to that of the unmodified hardened paste, modified cement pastes exhibited a 41% increase in its tensile strength. Previously, an increase of 28% in the tensile strength of the hardened cement pastes blended with nylon 66 nanofibers (N66 NFs) was observed [16]. Notably, this was the first time the role of N66 NFs increasing the tensile strength of cementitious materials became apparent. In the present work, with the addition of silica particles, the tensile strength showed the better result compared to that reported in the literature. A summary of these works, including the polymer components, electrospun nanofiber proportions, and mechanical results, is presented in Table 2.

In the case of compressive strength, an increase of 33% was observed with the addition of NS-N66 NFs, as compared to that of the unmodified paste (Figure 2a). In the previous study, the compressive strength only slightly increased, 8%, when similar proportions of N66 NFs were added to the pastes [16]. In addition, the toughness, calculated from the area under the constitutive curves of the hardened cement pastes based on the theory by Timoshenko and Gere [26], increased by 65% upon introducing NS-N66 NFs into the paste (Figure 2b). Based on our previous findings [16], the N66 NFs were ineffective in improving the compressive properties of cement. In contrast, the effectiveness of nylon 66 nanofibers modified by silica particles in improving the characteristics of cement paste in this study is apparent. We hypothesized that silica particles were attached alongside the nylon 66 nanofibers; thus, they filled the porous space inside the matrix and reacted with the CH created from the hydration process of the cement. As a consequence, a more compact structure was formed and a larger amount of CSH was created compared to that in the matrix of the unmodified paste; this, in turn, improved the compressive strength. The following sections discuss microstructure analyses to clarify this hypothesis.

### 3.2. Morphology and Microstructure of Nanofibers

**Figure 3:**
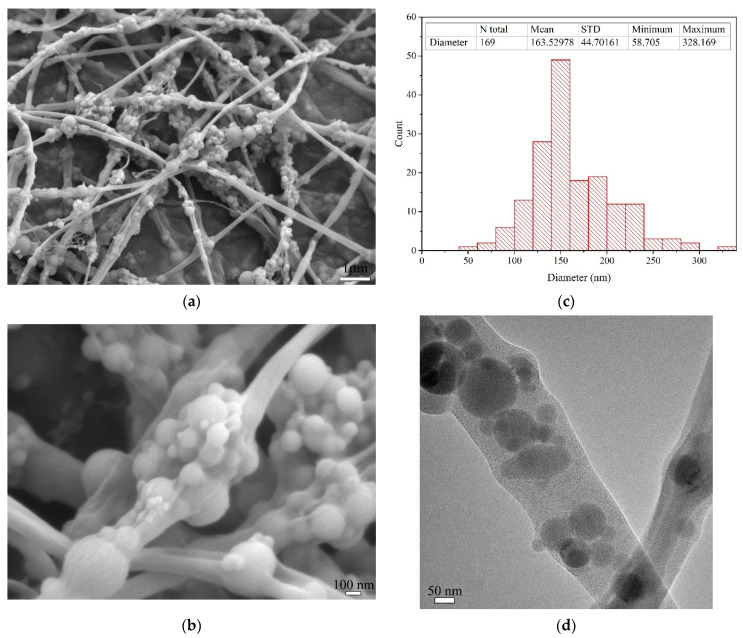
Morphological and microstructural characteristics of NS-N66 NFs. (**a**,**b**) SEM images of NS-N66 NFs at low and high magnification; (**c**) Diameter statistic; (**d**) TEM image of NS-N66 NFs.

Figure 3 presents the morphological and microstructural characteristics of the electrospun nylon 66 nanofibers modified by silica particles (NS-N66 NFs). In general, based on the SEM images in Figure 3a,b, the morphology of the fibers was observed, in which the silica particles were attached alongside the nylon 66 nanofibers. The resultant fibers contained thin nylon 66 nanofibers with a mean diameter of approximately 160–170 nm and silica particles with diameters of approximately 30–550 nm along the fiber axis. The size of the silica particles that exist along the fiber axis is almost consistent with that observed in the SEM image of NS, as presented above. However, despite the same electrospinning conditions, the diameter of the resultant nanofibers was slightly smaller than that observed in previous studies [16] due to the reduction of the nylon 66 content [27,28,29]. Figure 3d presents the TEM image of the modified electrospun nanofiber. From this observation, it is confirmed that there are only silica particles inside the nanofibers.

### 3.3. Microstructural Characteristic of Hardened Cement Pastes

**Figure 4:**
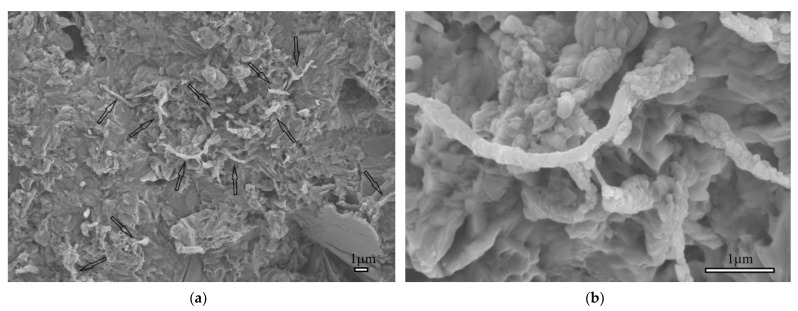
Microstructural characteristics of the cement matrix blended with NS-N66 NFs at low and high magnification (the arrows denote the fiber locations inside the cement matrix).

Figure 4 presents the microstructural characteristics of the electrospun nanofiber-blended cement matrix. In general, numerous nanofibers were observed inside the matrix. Figure 4a indicates that the nanofibers grew from the matrix and bridged the hydration products of the cement. This phenomenon showed the bridging and filling effects of the electrospun nanofibers inside the matrix. Moreover, compared with the smooth surface of the raw nylon 66 nanofibers containing silica particles along its axis, as presented above, the morphology of the nanofibers inside the cement matrix was completely transformed. The surface of the nanofibers became rough with granular structures, which was evidence of the pozzolanic reaction. It is suggested that nanosilica can consume the CH produced from the hydration of the cement, which creates more CSH in the final hydration products. In addition, some studies have confirmed the efficiency of silica-based additives in reducing the pore size of the cement matrix [3,4,5,6,7]. As a result, an improvement in the strength and impermeability of the cement matrix was observed. Thus, the silica particles attached alongside the nylon 66 nanofibers reacted with CH, the CSH was formed, gathered, and transformed the morphology of the nanofibers (Figure 4b). The occurrence of the pozzolanic reaction showed the good incorporation of the proposed electrospun nanofibers and cement materials. Above all, the SEM images indicate that the nylon 66 nanofibers modified by silica inside the cement matrix perform well. Specifically, owing to the bridging and filling effects of the proposed nanofibers, the microstructure of the cement matrix became more compact, resulting in improved mechanical characteristics.

### 3.4. XRD Analysis

**Figure 5:**
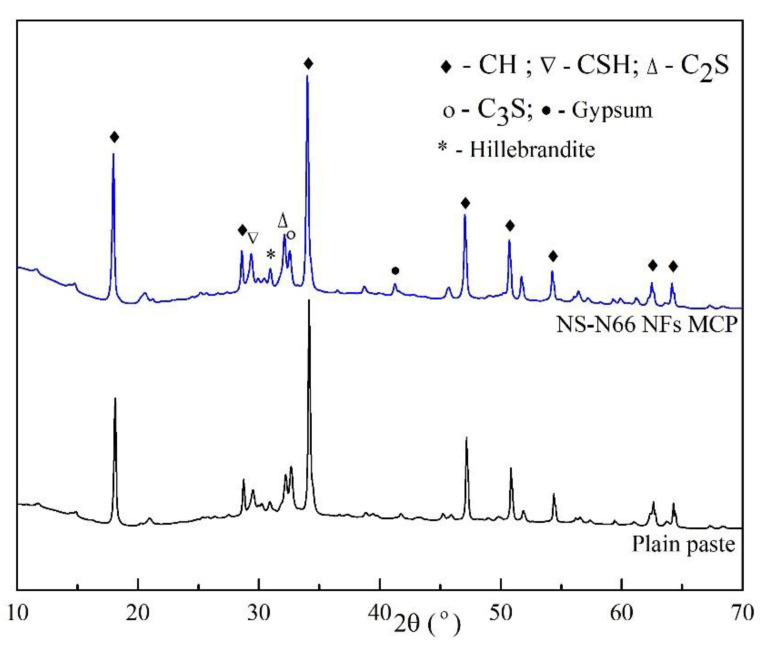
XRD patterns of the hardened cement pastes.

**Figure 6:**
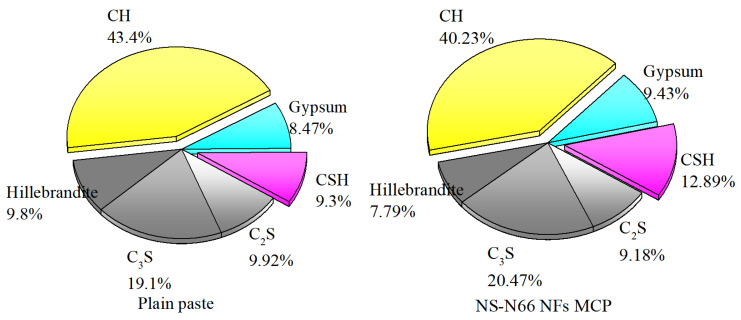
Component proportions of the cement matrices observed from the XRD results.

The XRD patterns of the plain cement and the electrospun nanofiber-blended cement are shown in Figure 5. Rietveld refinement [30] and phase determination were conducted based on the phase structure references of the hydration products, namely, calcium silicate hydrate (CSH), calcium hydroxide (CH), gypsum (CaSO_4_.2H_2_O), hillebrandite (Ca_2_SiO_3_(OH)_2_) and some dehydrated clinker grains, belite (C_2_S), and alite (C_3_S). The hydration products of all the samples were consistent and suitable with the results obtained from Jiang et al. [31] (Figure 5). Thus, the presence of the CH component can be correlated to the peaks between 17.94° and 18.11°, 28.62° to 28.74°, 34.02° to 34.15°, 47.03° to 47.17°, 50.73° to 50.84°, 54.29° to 54.39°, 62.51° to 62.59°, and 64.29° to 64.37° 2θ. The CSH component can be recognized by the peak between 29.40° and 29.46° 2θ. The peaks between 31.06° and 31.12°, 32.12° to 32.18°, 32.51° to 32.61°, and 41.10° to 41.20° 2θ corresponded to hillebrandite, belite, alite, and gypsum, respectively. Figure 6 shows the proportion of the hydration products calculated from the results of the Rietveld refinement using the Profex program. The CH content of the blended cement pastes decreased, while their CSH content increased. This suggests that the CH produced from the cement hydration process reacted with the NS particles attached along the nylon 66 nanofibers [31,32].

The decrease–increase tendency of the CH and CSH contents of the three samples was consistent with the order of the observations from the mechanical strength tests. The XRD analysis showed that the CH phase in the matrices was consumed. The increase in the CSH content was observed thanks to the pozzolanic reaction between the attached NS and the generated CH phase from the hydration processes.

### 3.5. Thermal Analysis

**Figure 7:**
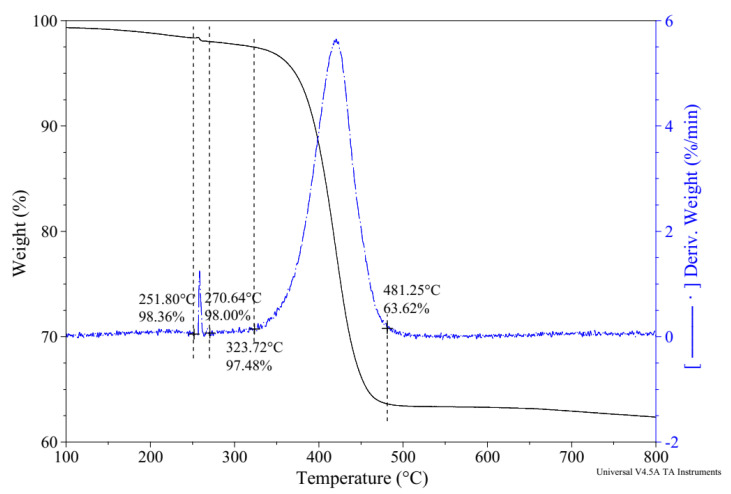
TGA-DTG results of NS-N66 NFs.

Figure 7 shows the TGA-derivative thermogravimetry (DTG) results of the NS-N66 NFs. The range from around 250 °C to 270 °C can be attributed to the evaporation of the adsorbed water from the nanosilica surface, and the range above 500 °C can be attributed to the condensation of the siloxyl groups. In addition, there was an incomplete decomposition process above 500 °C, that is, the silanol condensation was not complete at temperatures higher than 800 °C, which is in good agreement with the results from Das et al. [33]. Furthermore, the drop in mass from 310 °C to 480 °C can be attributed to the decomposition of the nylon 66 nanofiber, which is consistent with the results previously reported [16]. Thus, the decomposition of NS-N66 NFs occurred from 310 °C to 480 °C.

**Figure 8:**
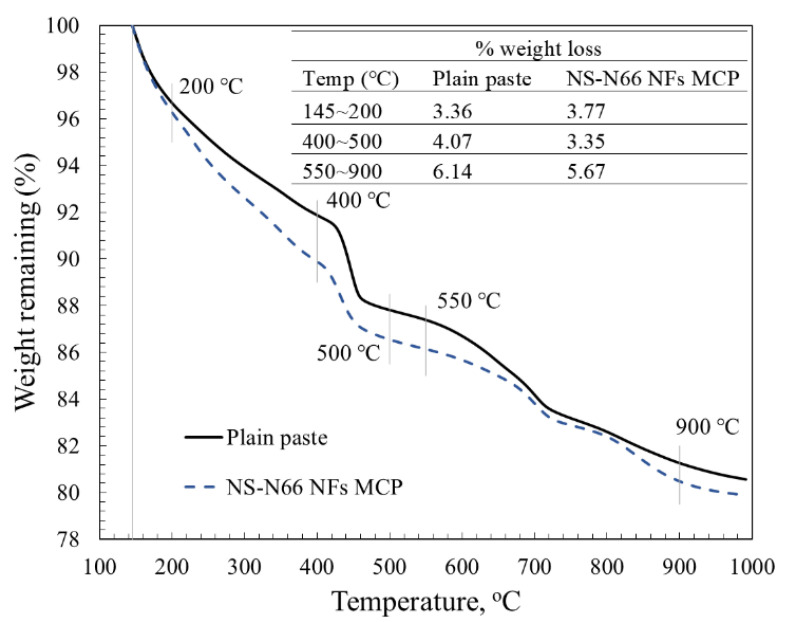
TGA-DTG results of the hardened cement pastes.

Figure 8 shows the TGA curves of the hardened cement pastes with and without the electrospun nanofibers after curing for 28 days. It is worth noting that the weight loss below 145 °C corresponds to the evaporation of free water inside the matrix when conducting the thermal analysis under nitrogen and free carbon dioxide conditions [34]. Therefore, the TGA results of the three samples were compared with 100% weight at 145 °C. The results also demonstrate the expected thermal behavior of the three main phases in the matrix: CH, CSH, and calcite. For instance, the CSH phase can be identified by the weight loss observed in the temperature range from 145 °C to 200 °C due to its dehydration process. The weight loss from 400 °C to 500 °C corresponds to the dehydration process of the CH phase, while that from 550 °C to 900 °C corresponds to the decarbonation of the calcite phase [34,35,36]. Chang et al. [37] reported that calcite was formed during the curing process when the CH phase gradually reacted with CO_2_ from the ambient environment. As can be observed from the TGA results, the CSH content from the nanofiber-blended cement pastes was higher than that of the plain paste, whereas that of CH was lower. It is also worth noting that despite the decomposition range of the CH phase being similar to the decomposition range of the electrospun nanofibers from 400 °C to 500 °C, as presented above, the CH content of the hardened modified cement pastes still decreased compared to that of the hardened plain paste, as indicated by TGA analysis. Consistent with the XRD results, these observations showed a decreasing tendency of the CH and CSH contents in the presence of NS-N66 NFs in the cement. Therefore, the attached silica particles on the nylon 66 nanofibers influenced the final product of the cement paste. Above all, these observations confirmed the initial hypothesis regarding the role of the pozzolanic agent in the attached silica particles.

## 4. Conclusions and Perspective

This study determined the influence of electrospun nylon 66 nanofibers containing silica particles on the microstructure and selected mechanical properties of the hardened cement pastes. The following conclusions were drawn:

An increase of 41%, 33%, and 65% in tensile strength, compressive strength, and toughness, respectively, was observed when modifying the cement pastes with the proposed nanofibers containing silica particles.

The nanosilica particles attached along the axis of the nylon 66 nanofibers were effective in increasing the generated CSH content from the pozzolanic reaction. In addition, the bridging and filling effects of the nanofibers inside the matrix were observed, which increased the mechanical strength of the hardened cement pastes.

The XRD and TGA results demonstrated the decrease–increase tendency of the CH and CSH contents when blending the cement with NS-N66 NFs. Therefore, the role of the attached silica particles is clarified.

Finally, electrospun nylon 66 nanofibers containing silica particles is a promising candidate for strengthening cementitious materials. It can be seen that an improvement in the mechanical properties of cement can lead to extending the durability and service life of concrete. However, for the practical application of this product, further research needs to be conducted to improve the performance of the composite cement fabrication process, as well as investigate the effectiveness of these nanofibers on other properties of cement paste and concrete. Additionally, the analysis of the life cycle and the life cycle cost of the concrete structure utilizing the proposed cement is necessary, which can evaluate the competitiveness of the proposed cement solution and help authorities in the decision-making process [38]. In addition, further comparative studies on the mechanical properties and microstructure of cement paste modified with silica particles, silica particles and nanofibers, and the proposed nanofibers containing silica particles are also interesting topics to analyze the performance of the proposed cement in this study.

## Figures and Tables

**Table 1:** Chemical composition and physical properties of the cement.

CaO	Al_2_O_3_	SiO_2_	SO_3_	MgO	Fe_2_O_3_	Ig. Loss	Specific Surface Area (cm^2^/g)	Compressive Strength, 28-Day (MPa)
61.33	6.40	21.01	2.30	3.02	3.12	1.40	2800	36

**Table 2:** Comparison of the mechanical strength results with [16] (the increase in %).

	Previous Study [16]		Present Study
	N66 NFs MCP (1)	N66 NFs MCP (2)	NS-N66 NFs MCP
Solvent (Volume %)	Formic acid: dichloromethane (4:1)	Formic acid: Chloroform (4:1)	Formic acid: Chloroform (4:1)
Polymer solution (Weight %)	N66 10 wt%: solvent 90 wt%	N66 10 wt%: solvent 90 wt%	NS 13 wt%: N66 7 wt%: solvent 80 wt%
Tensile strength	32	28	41
Compressive strength	6	8	33
Toughness	42	49	65

## Data Availability

The data presented in this study are available upon request from the corresponding author.
